# The influence of environmental factors related to Juvenile Dermatomyositis (JDM), its course and refractoriness to treatment

**DOI:** 10.1186/s42358-024-00408-5

**Published:** 2024-08-30

**Authors:** Clarissa C.M. Valões, Tamima M. Arabi, Alfésio L.F. Braga, Lúcia M.A. Campos, Nádia E. Aikawa, Kátia T. Kozu, Clovis A. Silva, Sylvia C.L. Farhat, Adriana M. Elias

**Affiliations:** 1 Hospital da Criança, Avenida Juca Sampaio, 67, Jacintinho Maceió, AL 57042-530 Brazil; 2 Rheumatology Unit, Child and Adolescent Institute, Hospital das Clínicas, Faculdade de Medicina FMUSP, Universidade de São Paulo, São Paulo, SP Brazil; 3 Environmental Epidemiology Study Group, Laboratory of Experimental Air Pollution, Faculdade de Medicina da Universidade de São Paulo, São Paulo, Brazil; 4 Rheumatology Unit, Child and Adolescent Institute and Rheumatology Division, Hospital das Clínicas HCFMUSP, Faculdade de Medicina, Universidade de São Paulo, São Paulo, Brazil

**Keywords:** Dermatomyositis, Tobacco smoking pollution, Occupational exposure, Air pollution, Environmental illness, Infancy

## Abstract

**Objective:**

To evaluate the influence of environmental factors and prematurity relating to juvenile dermatomyositis (JDM), its course and refractoriness to treatment.

**Methods:**

A case-control study with 35 patients followed up at a tertiary hospital and 124 healthy controls, all residents of São Paulo. Patients were classified according to monocyclic, polycyclic or chronic disease courses and refractoriness to treatment. The daily concentrations of pollutants (inhalable particulate matter-PM_10_, sulfur dioxide-SO_2_, nitrogen dioxide-NO_2_, ozone-O_3_ and carbon monoxide-CO) were provided by the Environmental Company of São Paulo. Data from the population were obtained through a questionnaire.

**Results:**

Fifteen patients had monocyclic courses, and 19 polycyclic/chronic courses. Eighteen patients were refractory to treatment. Maternal occupational exposure to inhalable agents (OR = 17.88; IC 95% 2.15–148.16, *p* = 0.01) and exposure to O_3_ in the fifth year of life (third tertile > 86.28µg/m^3^; OR = 6.53, IC95% 1.60–26.77, *p* = 0.01) were risk factors for JDM in the multivariate logistic regression model. The presence of a factory/quarry at a distance farther than 200 meters from daycare/school (OR = 0.22; IC 95% 0.06–0.77; *p* = 0.02) was a protective factor in the same analysis. Prematurity, exposure to air pollutants/cigarette smoke/sources of inhalable pollutants in the mother’s places of residence and work during the gestational period were not associated with JDM. Prematurity, maternal exposure to occupational pollutants during pregnancy as well as patient’s exposure to ground-level pollutants up to the fifth year of life were not associated with disease course and treatment refractoriness.

**Conclusion:**

Risk factors for JDM were maternal occupational exposure and exposure to O_3_ in the fifth year of life.

## Key points

• Maternal exposure to occupational pollutants during pregnancy was a risk factor for Juvenile Dermatomyositis (JDM).

• Exposure to ozone in the fifth year of life was found to be a risk factor for JDM.

• Exposure to air pollutants during the gestational period was not associated with JDM, its course or refractoriness.

## Introduction

Juvenile dermatomyositis (JDM) is the most common inflammatory myopathy of childhood [1], accounting for 80% of the cases [2]. The disease course has been described as monocyclic (defined as a one-course manifestation with no disease activity), chronic or persistent (patients who have maintained persistent disease activity for more than two years despite immunosuppressive treatment) and polycyclic (patients who had disease recurrence after clinical remission) [3]. Patients can be also classified according to response to treatment as refractory when they have some intolerance or inadequate responses to corticosteroids and at least one immunosuppressive drug [4].

Environmental factors act as a trigger on the immune system of genetically susceptible people and may cause the onset of JDM. Some triggers associated with the development of inflammatory myopathies have already been described, such as infections [5, 6], direct exposure to ultraviolet rays (UVR) and maternal exposure to inhalable elements [3, 7, 8].

In addition to these triggers, some factors influence the course of JDM, such as the presence of infection within the first six months of the disease, which increases the risk of its progression to a polycyclic course as opposed to a monocyclic one [9]. Moreover, Habers et al. showed that boys exposed to UVR one month before JDM diagnosis had an increased risk of progressing to a chronic course [3].

Air pollution consists of a mixture of gases and particles that includes carbon monoxide (CO), ozone (O_3_), nitrogen dioxide (NO_2_), sulfur dioxide (SO_2_), lead, toxic products from cigarette smoke, volatile organics, and particulate matter (PM) [10, 11]. Exposure to pollution was also associated with rheumatologic disease activity in children, including JDM patients [12]. Prematurity has also been described as a risk factor for several chronic morbidities such as hypertension, type 2 diabetes, cardiovascular diseases, obesity, psychiatric diseases [13] and pediatric lupus [14]. However, no study has assessed the association between exposure to air pollutants, as well as prematurity, and disease onset and evolution in juvenile myopathies.

Therefore, the aim of this study was to evaluate the influence of environmental factors and prematurity on development of JDM, its course and refractoriness to treatment.

## Methods

This is a case-control study with 35 JDM patients with ≤ 18 years old followed up at the Pediatric Rheumatology Unit, Child and Adolescent Institute, Faculdade de Medicina da Universidade de São Paulo (FMUSP), São Paulo (SP), Brazil, who met the classification criteria for the diagnosis of JDM [15]. The control group was composed of 124 healthy children and adolescents without chronic inflammatory diseases or lung diseases, in the same age group and sex as the group of patients. Children and their mothers during pregnancy had to be residents of cities in the metropolitan region of SP. Data collection was retrospective at pediatric outpatient clinics. Patients classified as monocyclic, polycyclic or chronic disease according to Habers et al. [3] and refractoriness to treatment according to Aggarwal et al. [4] were also assessed.

Free, Prior and Informed Consent was obtained from all participants and their legal guardians. The present study was approved by the Ethics Committee for Research Analysis of the FMUSP.

### Assessment of exposure to air pollutants

The São Paulo metropolitan area, which is 800 m above sea level, with 39 cities, 20 million inhabitants and an area of 946 km² is located in the southeast of the state of São Paulo. There are 22 automated pollution-monitoring stations spread around its area [14]. The São Paulo State Environmental Agency provided the daily measurement of inhalable PM_10_ (24-hour average), SO_2_ (24-hour average), NO_2_ (the highest hourly average), O_3_ (highest hourly average), CO (the highest 8-hour moving average). The average value of each pollutant measured in all seasons was adopted as representative of the exposure in the entire metropolitan region as used by Orione et al. and Conde et al. [8, 14] since air pollutant levels registered throughout each station were highly correlated.

Compared with the control group, an annual average of each pollutant was calculated considering date of birth of each child as a starting point. For the assessment of the two years before and after the onset of symptoms, an estimate of the date of onset of symptoms reported at the first consultation was performed. For the evaluation of the gestation period, we used the 270 days that preceded the date of birth, and for the evaluation by quarter, we considered 90 days in each trimester.

For disease course analysis, patients with a polycyclic and chronic course remained together in the same group as new evidence suggests that patients with a polycyclic course may maintain subclinical activity undetectable by current disease assessment methods [16].

### Structured questionnaire

**Fig. 1:**
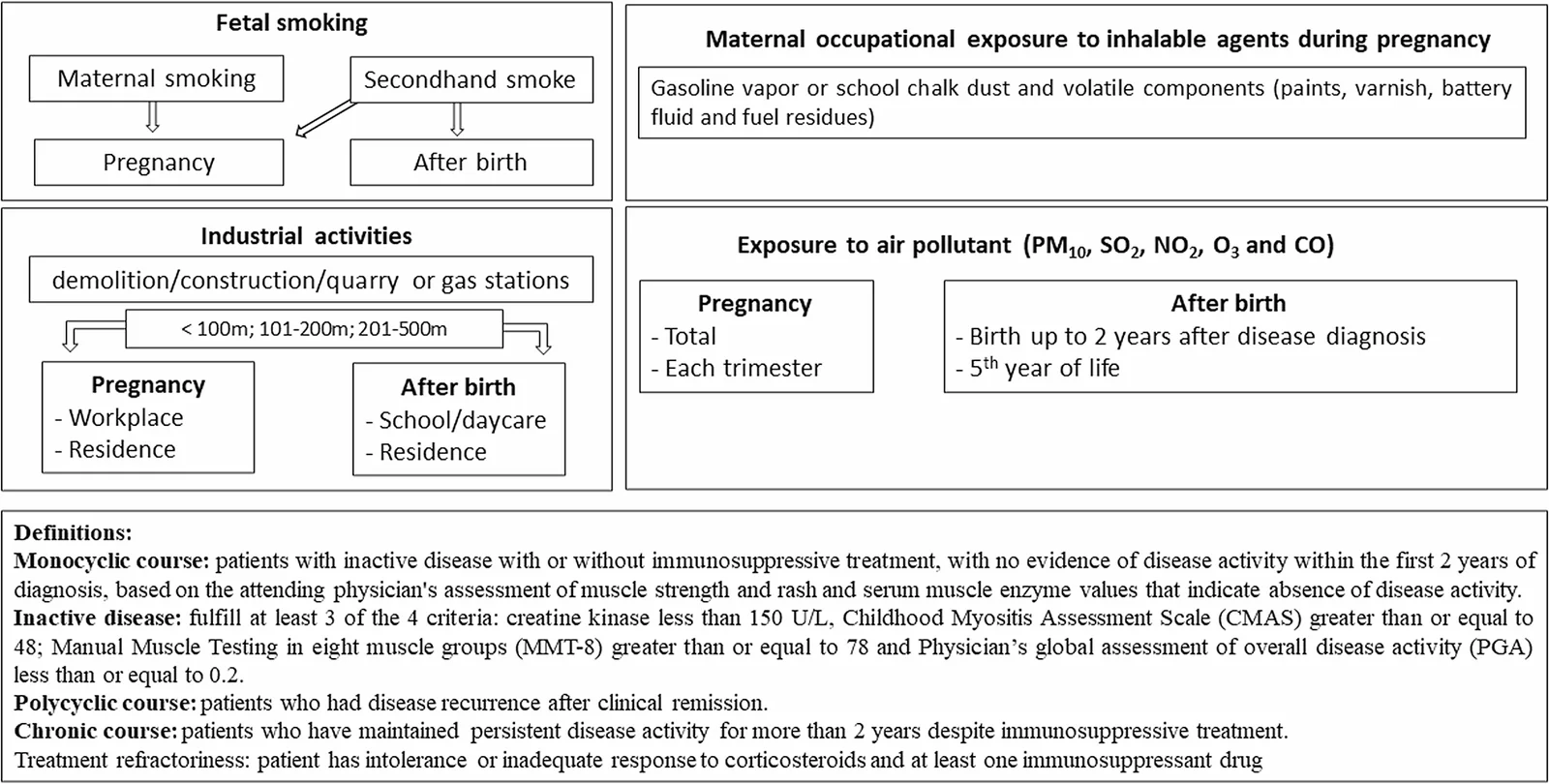
The main variables studied in the analysis between the groups (JDM, control group, monocyclic, polycyclic/chronic groups, refractory and non-refractory groups)

Data from JDM patients and healthy controls were obtained from their mothers through the modified structured questionnaire used by Guimarães et al. [17], with the objective of obtaining data referring to period of pregnancy and from birth to onset of disease symptoms. The independent variables studied were obtained from the questionnaire. The main variables studied in the analysis between the group with JDM and the control group, further distinguishing monocyclic and polycyclic/chronic groups, as well as refractory and non-refractory groups are (Fig. 1):

- Fetal smoking (maternal and/or secondhand smoking);
- Maternal occupational exposure to inhalable agents: gasoline vapors or school-chalk dust and volatile components (paints, varnish, battery fluid and fuel residues);
- Information of industrial activities (demolition/construction/quarry) or gas stations within a radius of up to 500 m from the residence/workplace/daycare or school during JDM patients’ and controls’ pregnancies and after birth until JDM diagnosis
- Indicators of exposure to air pollutants during pregnancy, in each quarter of pregnancy and birth up to two years after disease diagnosis
- Prematurity, defined as birth before 37 weeks of gestational period, weight gain between 11–16 kg during pregnancy and weight at birth were also assessed [18].

### Statistical analysis

Continuous variables were presented in terms of measures of central tendency and dispersion (mean ± standard deviation or median and variation) and categorical variables in absolute and relative values in the case and control groups. Data were compared by Student’s t-test or Mann-Whitney test for continuous variables, and by chi-square test or Fisher’s exact test for categorical variables.

The average concentrations of tropospheric pollutants were calculated in tertiles for each participant in each of the periods evaluated. In the analysis between the JDM group and the control group, the study assessed exposure to ground-level pollutants up to the fifth year of life as median and mean age at diagnosis was about five years old. When comparing disease course and refractoriness to treatment, we considered exposure to ground-level pollutants up to two years after diagnosis, the time required to classify the patient according to the course of disease.

Multilevel binary logistic regression was used to identify risk factors for the diagnosis of JDM, for each course of the disease, and for disease refractoriness to initial treatment. In the multilevel model, variables with a significance level lower than or equal to 20% in the univariate model were included as independent variables. In the final multiple models, the variables that presented a level lower than 5% of significance in multilevel models were included for final analysis.

Model results were presented as odds ratios (OR) and 95% confidence intervals (CI). In all statistical tests, the significance level was set at 5% (*p* < 0.05).

The program used for statistical analysis was IBM-SPSS-23.

## Results

The kappa coefficient for the test-retest was 0.81, meaning high agreement between mothers’ answers and reliability of the instrument used. Table 1 shows the demographic data of the groups analyzed (JDM, monocyclic, polycyclic/chronic and control groups).

**Table 1:** Demografic data of the analyzed groups (JDM, monocyclic, polycyclic/chronic and control groups)

	JDM group N = 35	Control group n = 124	Monocyclic course n = 15	Chronic or polycyclic course n = 19	Monocyclic versus chronic or polycyclic ***p***	JDM versus control group ***p***
Female subjects*	19 (54%)	71 (57%)	8 (53%)	10 (52%)	1.00	0.85
Mean chronological age in years (SD)	13.8 (4.9)	11.2 (4.2)	–	–	–	0.17
Mean age at diagnosis in years (SD)	5.8 (±2.5)	–	6.25 (±2.5)	5.7 (±2.4)	0.96	–
Refractoriness to treatment	18 (51%)	–	5 (33%)	12 (63%)	0.17	–

*JDM* juvenile dermatomyositis, *SD* standard deviation

*A female subject, classified as refractory, had incomplete chart data 2 years after disease diagnosis, making it impossible to classify her as to the course of the disease. She was excluded from the analysis of this classification

Smoking, occupational exposure and prematurity were associated with JDM in the univariate analysis of logistic regression models; no association was found between child’s secondhand smoking and JDM (Table 2).

**Table 2:** Gestational, perinatal-related factors and after birth factors as risk for JDM in univariate logistic regression models

Variables	JDM (%)	Control group (%)	***p***	Univariate logistic regression model		
During gestation				OR	CI (95%)	*P*
Fetal smoking (mother and secondhand)	48.5	21	0.003*	3.55	1.58–7.96	0.002*
Maternal occupational exposure to one of the inhalable agents	26.6	5.94	0.024*	5.76	1.40–23.6	0.015*
After birth						
Prematurity	14	4	0.04*	3.97	1.08–14.59	0.04*
Child’s secondhand smoke	33.3	23.4	0.26	1.93	0.79–4.67	0.15

*OR* odds ratio, *CI* confidence interval, *statistically significant, *JDM* juvenile dermatomyositis

JDM patients were more frequently exposed to factories or quarries at less than 500 m from the residential address compared to the control group (48.5% vs. 13.7%, *p* = <0.001). The same was found for the presence of factories/quarries at a distance < 500 m from work address during pregnancy (35.7% vs. 10%, *p* = 0.02), the address of daycare/school (20.8% vs. 6.45%, *p* = 0.04) and of residential address after birth (46.66% vs. 10.48%, *p* < 0.001). In the univariate analysis, the presence of emitting sources of pollutants farther than 200 m from work address and/or home during pregnancy was associated with JDM (OR = 2.36; IC95% 1.04–5.33; *p* = 0.04). Distances less than 100 m and between 100–200 m were not associated [(OR = 1.33; CI95% 0.50–3.55, *p* = 0.57) and (OR = 1.35; CI95% 0.53–3.41; *p* = 0.53)]. But distance farther than 200 m from the sources of pollutants and the address of daycare/school showed a negative association with JDM (OR = 0.24; CI 95% 0.05–0.74; *p* = 0.013).

There was no difference between the two groups in terms of weight at birth (88% vs. 89% of patients were born weighing between 2500 g and 4000 g, *p* = 0.73, respectively) and weight gain during pregnancy (50% vs. 48.39% of the mothers showed ideal weight gain, *p* = 0.49, respectively).

Exposure to O_3_, NO_2_ and SO_2_ during pregnancy, PM_10_ and SO_2_ in the first quarter, NO_2_, SO_2_ and CO in the third quarter, showed association with JDM with a *p* < 0.20 in the univariate analysis. In the multivariate analysis just between the pollutants during pregnancy, O3 exposition (second tertile 77.84–84.94 μg/m^3^, OR = 0.15, IC95% 0.04–0.54, *p* = 0.004) and PM_10_ (second tertile 38.69–49.41 μg/m^3^, OR = 3.95, IC95% 1.2–13.02, *p* = 0.024) persisted significantly associated with JDM.

Exposure to O_3_, PM_10_, SO_2_ and CO in the fifth year was associated with JDM in the univariate analysis and exposure to O_3_ was associated in the multivariate analysis (third tertile > 86.28 μg/m^3^, OR = 5.43; IC95% 1.29–22.7; *p* = 0.02).

Table 3 presents the multilevel logistic regression performed to identify possible risk factors for JDM diagnosis during gestation using all variables in the univariate analysis that showed *p* < 0.20 and the tropospheric pollutants that showed *p* < 0.05 in the multivariate analysis. In this analysis, maternal exposure to some occupational pollutants during pregnancy was found to be significantly associated with JDM. Table 4 presents the final multilevel analysis with the post-birth variables associated with JDM. In this analysis, exposure to O_3_ was found as a risk factor for JDM in the fifth year of life and sources of pollutants farther than 200 m from daycare/schools was a protective factor.

**Table 3:** Gestational and perinatal-related factors and environmental factors during gestation as risk factors for JDM in multilevel logistic regression models

Independent variables	OR	CI (95%)	***P***
During gestation			
O_3_ (μg/m^3^)			
Second tertile (77.84–84.94)	0.65	0.07–5.81	0.70
Third tertile (>84.95)	0.26	0.05–1.48	0.13
PM_10_ (μg/m^3^) in the 1st trimester of pregnancy			
Second tertile (38.69–49.41)	2.41	0.18–32.08	0.50
Third tertile (>49.41)	6.51	0.98–43.42	0.05
Prematurity	0.20	0.01–2.69	0.22
Maternal exposure to tobacco during pregnancy	3.34	0.06–1.12	0.07
Maternal exposure during pregnancy			
Exposure to inhalable pollutants at work or/and residence	3.40	0.87–13.25	0.08
Maternal occupational exposure to inhaled particles or volatile components	17.88	2.15–148.16	0.01*

*OR* odds ratio, *CI* confidence interval. * statistically significant, *JDM* juvenile dermatomyositis

**Table 4:** Gestational and perinatal-related factors and environmental factors at fifth year of life as risk factors for JDM in multiple logistic regression models

Independent variables	OR	CI (95%)	p
Fifth year of life			
O_3_ Third tertile (> 86,28µg/m^3^)	6.53	1.60–26.77	0.01*
Exposure to inhalable pollutants			
Daycare/school < 100 m	0.08	0.005–1.42	0.86
Daycare/school 100–200 m	0.37	0.09–1.57	0.18
Daycare/school > 200 m	0.22	0.06–0.77	0.02*
Prematurity	1.47	0.24–9.12	0.68
Secondhand smoking	0.23	0.62–7.47	2.14

*OR* odds ratio, *CI* confidence interval. * statistically significant, *JDM* juvenile dermatomyositis

### Monocyclic and polycyclic/chronic course analysis

Regarding maternal smoking during pregnancy, there was no difference between the two groups (6.6% vs. 17.6%, *p* = 0.60, respectively). There was also no difference when evaluating the fetal smoking (40% vs. 59%, *p* = 0.48), as well as the child’s exposure to secondhand smoking after birth (37% vs. 40%, *p* = 1.00). In the univariate analysis in the logistic regression models, there was no association between maternal smoking (OR = 0.33; CI95% 0.03–3.31; *p* = 0.37) or secondhand smoking during pregnancy (OR = 0.33; CI95% 0.11–1.92; *p* = 0.29). There was no difference when assessing maternal occupational exposure during pregnancy (25% vs.17%, *p* = 1.00). Regarding the presence of sources emitting inhalable pollutants close to home (address during pregnancy and after birth), close to the mother’s work address during pregnancy and close to daycare/school, there was no statistical difference between the two groups (*p* > 0.05). In the univariate analysis, we found no association in the assessment of the presence of factories (OR = 2.86, CI95% 0.67–12.11, *p* = 0.15) and gas stations (OR = 0.28, CI95% 0.06–1.20, *p* = 0.09) near the address of the pregnancy. Prematurity was not different between the two groups (27% vs. 5%, *p* = 0.15, respectively) and was not associated with the disease course in the univariate analysis (OR = 0.153, CI95% 0.01–1.55; *p* = 0.19). Weight at birth (92.30% of vs. 83.55% of patients were born weighing between 2500 g–4000 g, *p* = 0.59) and weight gain during pregnancy (54.54% vs. 42.85% of mothers had an ideal weight gain, *p* = 1.00) showed no difference.

The pollutants that showed *p* < 0.20 in the univariate analysis were O_3_, PM_10_, NO_2_ during pregnancy, NO_2_ and CO in the first trimester, PM_10_, NO_2_, in the second trimester and O_3_ and NO_2_ in the third trimester. Only exposure to NO_2_ in the third trimester was associated with the monocyclic course in the multivariate analysis of pollutants.

Table 5 presents the final multivariate analysis performed to identify possible risk factors for the monocyclic course, including gestational and fetal factors. In this analysis, no variable was associated with disease course.

**Table 5:** The final multivariate analysis performed to identify possible risk factors for the monocyclic course, including gestational and fetal factors

Independent variables	OR	CI (95%)	p
During gestation			
NO_2_ (μg/m^3^)			
First tertile (<84.83)	–	–	0.21
Second tertile (84.83–101.64)	1.01	0.04–26.99	1.00
Third tertile (>101.65)	10.18	0.29–362.8	0.20
Prematurity	0.74	0.04–12.65	0.84
Exposure to inhalable pollutants at residence during pregnancy	3.03	0.33–27.45	0.32
Presence of gas stations near the address of the pregnancy	0.08	0.004–1.69	0.10

*JDM* juvenile dermatomyositis, *NO*_*2*_ nitrogen dioxide, *OR* odds ratio, *CI* confidence interval. (95%)

Exposure to O_3_ and CO (second tertile 1.32–2.44ppm, OR = 7.87, IC95% 1.10–56.12; *p* = 0.04) two years before diagnosis, PM_10_, NO_2_ one year before the diagnosis, PM_10_ and NO_2_ (second tertile 68.73–96.75µg/m^3^, OR = 0.12; IC95% 0.01–0.97, *p* = 0.047) one year after diagnosis and NO_2_ and CO two years after diagnosis was associated with monocyclic course in univariate analysis, but none remained association in multivariate analysis with disease course (*p* > 0.05).

### Refractory and non-refractory analysis

Mean age at diagnosis was comparable between patients with refractory and non-refractory disease (6.3 ± 2.8 years vs. 5.3 ± 2.1 years, *p* = 0.37), as well as mean age at diagnosis and the frequency of female subjects (50% vs. 58.8%, *p* = 0.74). Mean age at symptom onset was 4.6 (±1.95) years and median of 4.5 years in the group of non-refractory patients and 5.6 (±2.7, *p* = 0.20) years and median of 5.3 years in refractory patients (*p* = 0.20).

Regarding maternal smoking during pregnancy, there was no difference between the two groups (11.7% vs. 12.5%, *p* = 1.00). There was also no difference when assessing maternal secondhand smoking (56.2% vs. 41.2%, *p* = 0.49), as well as in the analysis of child exposure to secondhand smoking (30.7% vs. 48.8%, *p* = 0.70). In the univariate analysis, maternal smoking (OR = 0.93; CI95% 0.11–7.55; *p* = 0.95), maternal secondhand smoking during pregnancy (OR = 0.54; CI95% 0.14–2.17; *p* = 0.39) and the child’s exposure to secondhand smoking (OR = 1.69; 95% CI95% 0.35–8.22; *p* = 0.52) were not associated to refractoriness and were not used in the multivariate evaluation (*p* > 0.20).

No difference was found regarding maternal work during pregnancy between the two groups (16.6% vs. 33.3%, *p* = 0.60). In the univariate analysis, maternal occupational exposure (OR = 0.87; CI95% 0.13–11.50; *p* = 0.87) was not associated with refractoriness.

The presence of factories/quarries and gas station at less than 500 m from the home during pregnancy and after birth, from the maternal work address during pregnancy, and from the daycare/school were not different between the two groups (*p* > 0.05). The univariate analysis of the presence of gas stations at less than 500 m from the mother’s work address during pregnancy showed OR = 0.07; CI95% 0.005–1.06, *p* = 0.055, the other variables showed *p* > 0.20. Prematurity (23.5% vs. 5.8%, *p* = 0.33, respectively), weight at birth between 2500 g–4000 g (86.61% vs. 91.67%, *p* = 1.00, respectively), ideal maternal weight gain during pregnancy (50% vs. 50%, *p* = 1.00, respectively) showed no difference between refractory and non-refractory group. In the univariate analysis, prematurity was not associated with refractoriness to treatment (OR = 4.92; CI95% 0.49–49.6, *p* = 0.18).

The tropospheric pollutants that showed *p* > 0.20 in univariate analysis were O_3_, PM_10_, NO_2_ during pregnancy, PM_10_ in the first trimester, O_3_, PM_10_, NO_2_, CO in the second trimester and PM_10_ and SO_2_ in the third trimester. None of them were associated with refractoriness in the multivariate analysis.

The variables involved in fetal exposure that were associated with refractoriness (*p* < 0.20) in the univariate analysis were the presence of gas stations less than 500 m from the mother’s work address during pregnancy and prematurity. However, in the multivariate analysis, there was no association with refractoriness to treatment (*p* = 1.00).

Further multivariate analysis was not performed as there was no association between tropospheric pollutants and refractoriness to treatment in the univariate analysis and the only variable associated with postnatal exposure that could be included in the analysis was prematurity (*p* = 0.18).

## Discussion

For the first time, the association of exposure to atmospheric pollutants and gestational factors was investigated in relation to the course and refractoriness of JDM. Prematurity, maternal exposure to occupational pollutants during pregnancy as well as patient’s exposure to ground-level pollutants up to the fifth year of life were not associated with disease course or treatment refractoriness. Nevertheless, maternal occupational exposure to inhalable agents and to O_3_ in the fifth year of life were identified as risk factors to JDM development.

Over the last 30 years we have seen an increase in the frequency of autoimmune diseases. The recent increase in these diseases in industrialized countries has brought up attention to the possible causes that contributed to this. Considering that genetic factors are constant, environmental factors have become the targets of investigation. In fact, in recent decades, there have been significant changes in Western eating habits, exposure to environmental pollution and an increase in stress levels, and these factors are currently the focus of more recent studies [19].

Among the environmental factors associated with the development of autoimmune diseases, we can mention exposure to chemicals, which can be grouped into persistent organic pollutants, toxic solvents (such as acetone, butanol, toluene, benzene and methanol, found in paints, glues and paint strippers), toxic heavy metals and endocrine disruptors. These chemical compounds can induce oxidative stress, T cell dysregulation and changes in cell-mediated response [20].

Maternal occupational exposure to inhalable particles or volatile compounds was found to be a risk factor for the development of pediatric lupus [14] and JDM [8]. In the present study, maternal occupational exposure was a risk factor for JDM but there was no association of this exposure with the course or refractoriness to disease.

However, in our multivariate analysis, smoking was not a risk factor for JDM and was not associated with disease course and treatment refractoriness. Differently of observed by Orione et al. in their series found fetal exposure to smoking as a risk factor for the development of JDM [8]. The sample of the work carried out previously in the group and published by Orione et al. presented 20 patients with JDM and 56 controls [8]. The present study added 15 patients to the previous series, and the increase in the number of participants may have been the reason we presented different statistical results.

Scalabrini et al. [6] carried out a study involving 107 patients with a probable or definitive diagnosis of juvenile inflammatory myopathy (Bohan and Peter criteria) who lived in 17 American states and in 9 climactic regions. The study used a questionnaire to assess exposure to environmental factors by patients, who were grouped according to the presence of autoantibodies (anti-TIF-1, anti-NXP2, anti-MDAS and negative antibodies) and the clinical subgroups (JDM, juvenile polymyositis, juvenile connective tissue myositis). In the aforementioned study, no associations were found between prematurity, exposure to maternal, paternal or other person smoking during pregnancy and clinical or serological subtype. These results were similar to ours. Other forms of exposure to environmental pollutants were not evaluated.

Prematurity has been described as a risk factor for the development of childhood-onset systemic lupus erythematosus [14] and juvenile idiopathic arthritis [21]. The authors postulate that association between preterm delivery (before 37 weeks of gestational age) and pediatric chronic diseases and autoimmune diseases is related to epigenetic mechanisms [13, 14]. In the present study, prematurity was not a risk factor for the development of JDM and was not associated with course or refractoriness to treatment.

Air pollutants have been linked to many harmful effects on human health. PM is one of these pollutants and originates mainly from the burning of fuels and is therefore related to urban areas due to vehicular traffic [10]. A recent study found a positive association between the incidence of autoimmune rheumatic diseases (including dermatomyositis) and PM, but only the adult population was studied using data from a cohort from the province of Quebec in Canada. In the same study, no association was found between immune-mediated rheumatic diseases and ozone [22]. Another study also carried out in Canada also found an association between PM_2,5_ and immune-mediated rheumatic diseases, but the same group found no clear association between NO_2_ and these diseases [23, 24]. In our series, exposure to O_3_ in the fifth year of life was a risk factor for JDM. We found no association between tropospheric pollutants and the course of the disease or refractoriness to treatment.

In the multivariate analysis of tropospheric pollutants, we found a negative association between O_3_ during pregnancy and JDM, but in the final multivariate analysis of gestational risk factors, this association did not remain. Kuo et al. found a negative association in the multivariate analysis between exposure to O_3_ during pregnancy and another inflammatory disease (Kawasaki disease) [25]. The author postulates that the possible explanation for this is that the effect of exposure to O_3_ is dose-dependent: high doses stimulate intense oxidative stress, resulting in an inflammatory response and tissue damage, while low concentrations of O_3_ induce moderate oxidative stress that would activate protective antioxidant pathways. However, more clinical and experimental studies are needed on the role that O_3_ can play in pregnancy and in the development of autoimmune and inflammatory diseases. In our series, exposure to tropospheric pollutants was not a risk factor for the course and refractoriness to treatment.

One of the confounding factors that may have interfered with the results was UVR. UVR depends on the concentration of ozone, the geographical position of the location, altitude, time of day, season of the year, atmospheric conditions and type of surface, with all these parameters being used to calculate the UVR index [26]. UVR has already been described as a risk factor for the evolution of the disease to the chronic course in boys exposed one month before diagnosis when compared with the polycyclic course [3]; it was also associated with the clinical phenotype in Caucasian individuals [27], disease activity in a population of pediatric and adult patients diagnosed with dermatomyositis [26] and disease severity when assessing the presence of calcinosis [7].

The present study did not assess exposure to UVR due to the lack of available data on this kind of exposure. Although UVR data refers to pollution in its calculation, it is interesting that both variables (UVR and atmospheric pollutants) are analyzed together, since the interaction between radiation and pollution is complex. For example, the formation of ozone in the troposphere occurs through chemical reactions between NOx and volatile organic compounds in the presence of solar radiation, and the NO, emitted in the burning of fuels also acts on the consumption of tropospheric ozone, causing low ozone concentrations near traffic lanes.

In addition, this interaction during the gestational period seems to be distinct from the interaction after birth, as occurs with exposure to O_3_. This reinforces the need for further research involving this phase, which is so important for human development. This favors the current findings that epigenetic mechanisms play an important role in determining the health-disease process.

Other limitations of the study were: the retrospective data collection; the sample size of patients, since only those who lived in the metropolitan region of São Paulo and whose mothers had lived in the same region during the gestational period were included; the possibility of recall bias, since we used the questionnaire; and the form of pollution monitoring that does not allow us to assess exposure to environmental pollutants individually. At the moment, new research is underway involving environmental factors and JDM, comparing the Brazilian population with the North American population.

## Conclusion

Maternal exposure to occupational pollutants during pregnancy was a risk factor for JDM and showed no association with disease course or treatment refractoriness. Exposure to O_3_ in the fifth year of life was found to be a risk factor for JDM, and the presence of sources of inhalable pollutants at a distance of at least 200–500 m from the daycare/school was found to be a protective factor. Exposure to air pollutants/cigarette smoke/other sources of inhalable pollutants in the mother’s address of residence and work during the gestational period was not associated with JDM, its course or refractoriness.

## Data Availability

The datasets used and/or analysed during the current study are available from the corresponding author on reasonable request.
